# Effects of rmBMP-7 on Osteoblastic Cells Grown on a Nanostructured Titanium Surface

**DOI:** 10.3390/biomimetics7030136

**Published:** 2022-09-16

**Authors:** Leonardo Raphael Zuardi, Fabíola Singaretti de Oliveira, Roger Rodrigo Fernandes, Maria Paula Oliveira Gomes, Silvia Spriano, Antonio Nanci, Paulo Tambasco de Oliveira

**Affiliations:** 1 Department of Basic and Oral Biology, School of Dentistry of Ribeirão Preto, University of São Paulo, Ribeirão Preto 14040-904, SP, Brazil; 2 Department of Oral and Maxillofacial Surgery and Periodontics, School of Dentistry of Ribeirão Preto, University of São Paulo, Ribeirão Preto 14040-904, SP, Brazil; 3 Department of Applied Science and Technology, Politecnico di Torino, 10129 Torino, Italy; 4 Faculté de Médecine Dentaire, Université de Montréal, Montreal, QC H3T 1J4, Canada

**Keywords:** BMP-7, osteoblast, titanium, nanotopography

## Abstract

This study evaluates the effects of the availability of exogenous BMP-7 on osteoblastic cells’ differentiation on a nanotextured Ti surface obtained by chemical etching (Nano-Ti). The MC3T3-E1 and UMR-106 osteoblastic cell lines were cultured for 5 and 7 days, respectively, on a Nano-Ti surface and on a control surface (Control-Ti) in an osteogenic medium supplemented with either 40 or 200 ng/mL recombinant mouse (rm) BMP-7. The results showed that MC3T3-E1 cells exhibited distinct responsiveness when exposed to each of the two rmBMP-7 concentrations, irrespective of the surface. Even with 40 ng/mL rmBMP-7, important osteogenic effects were noticed for Control-Ti in terms of cell proliferation potential; *Runx2*, *Osx*, *Alp*, *Bsp*, *Opn*, and *Smad1* mRNA expression; and in situ ALP activity. For Nano-Ti, the effects were limited to higher *Alp*, *Bsp*, and *Opn* mRNA expression and in situ ALP activity. On both surfaces, the osteogenic potential of UMR-106 cultures remained unaltered with 40 ng/mL rmBMP-7, but it was significantly reduced when the cultures were exposed to the 200 ng/mL concentration. The availability of rmBMP-7 to pre-osteoblastic cells at the concentrations used alters the expression profile of osteoblast markers, indicative of the acquisition of a more advanced stage of osteoblastic differentiation. This occurs less pronouncedly on the nanotextured Ti and without reflecting in higher mineralized matrix production by differentiated osteoblasts on both surfaces.

## 1. Introduction

During the integration of biomaterials with bone tissue, different pre-existing and/or de-novo-secreted molecules modulate the activities of bone cells, resulting in the synthesis of a new mineralized extracellular matrix, its maturation, and remodeling. Based on this, strategies for developing and improving alloplastic implants take into account the availability of key molecule(s) on their surfaces, aimed at accelerating bone growth and increasing bone mass at the implant site, especially in anatomical regions with low bone density [1,2].

Bone morphogenetic proteins (BMPs) constitute a class of growth factors belonging to the transforming growth factor beta (TGF-β) superfamily with recognized stimulatory effects on osteoblast activity during the formation and repair of bone tissue [3]. Therefore, BMPs are considered good candidates for the surface functionalization of osseointegrated implants. Among the different types of commercially available recombinant BMPs, only BMPs 2 and 7 have been approved by the Food and Drug Administration (FDA) for use in therapeutic strategies in humans [4]. Based on the available studies of the use of BMP-2 and BMP-7 in bone defects and/or implant sites by means of gene or protein delivery, the results for BMP-2 have been shown to be consistent for the stimulation of bone repair [5,6] except in situations of exaggerated inflammation [7]. On the other hand, the stimulatory effects related to the use of BMP-7 remain controversial and a subject of debate [3,8,9,10]. It has been suggested that BMP-2 and BMP-7 have differing effects on bone cell differentiation and that BMP-2 is essential for postnatal bone formation [11]. The controversy surrounding BMP-7′s effects on bone repair, particularly when it is associated with biomaterials, could be related to the BMP-7 concentration in the extracellular milieu and the differentiation stage of the target osteoblastic cell as well as the physicochemical characteristics of the biomaterial surface, which determine the release profile of the adsorbed and/or grafted molecule and can modulate the cell’s responsiveness to it [3,12,13]. Here, we opt to investigate the effects of BMP-7 using conventional 2D osteogenic cell culture models.

Over the past two decades, the development of osseointegrated implant surfaces has established their structuring at the micron scale as a fundamental modification for stimulating bone deposition in direct contact with the implant. Furthermore, nanoscale surface modifications have also been applied due to their known beneficial effects on bone cells and tissue repair [14,15]. For example, the surface nanotopography of titanium (Nano-Ti), created by a chemical treatment based on sulfuric acid (H_2_SO_4_) and hydrogen peroxide (H_2_O_2_), stimulates the initial secretion of bone sialoprotein (BSP) and osteopontin (OPN)—two important osteoblast markers—and promotes increased mineralized matrix formation in vitro and in vivo [16,17,18], an effect that involves the activation of the BMP-2 and α1β1 integrin signaling pathways [12,19,20]. In addition to these effects, nanotopography and multiscale roughness result in a larger surface area, with a potentially major impact on the capability to retain endogenous molecules by adsorption and/or to favor the functionalization of exogenous molecules, ultimately modulating specific cell functions [21,22,23].

Therefore, the present study aims to evaluate the effects of the extracellular availability of BMP-7 during the acquisition of the osteogenic phenotype by cultured osteoblastic cells grown on two Ti surfaces with distinct topographical features—Polished (Control-Ti) and Nano-Ti. The effects of an eventual release of grafted BMP-7 by a functionalized surface were mimicked by adding BMP-7 into the culture medium. The null hypothesis was that BMP-7′s effects on osteoblastic cells, particularly on their osteogenic potential, were not modulated by the surface topography of Ti at the nanoscale.

## 2. Materials and Methods

### 2.1. Preparation of Titanium (Ti) Surfaces

Commercially pure grade 2 titanium (Ti) discs (13 mm in diameter and 2 mm thick) (Realum, São Paulo, SP, Brazil) were polished using 300, 400, and 600 grit silicon carbide papers and cleaned by sonication (Control-Ti group). To obtain the Nano-Ti surface, Ti discs were etched in a solution of equal volumes of concentrated H_2_SO_4_ (95–97%) and 30% H_2_O_2_ for 4 h (10 mL/disc) [24] in a thermostatic ice bath under constant agitation. Control-Ti and Nano-Ti discs were washed in distilled water, air-dried, and autoclaved prior to the biological assays.

### 2.2. Osteogenic Cell Cultures

The pre-osteoblastic MC3T3-E1 cells, subclone 14 (CRL-2594, ATCC, Manassas, VA, USA), were used for the 5-day experiments, except for the mineralization assay (see below). Firstly, they were grown in 75 cm^3^ culture flasks (Corning Inc., Kennebunk, ME, USA) with 16 mL of α-MEM culture medium (Invitrogen, Carlsbad, CA, USA), 10% fetal bovine serum (Invitrogen), and 1% penicillin–streptomycin (Sigma-Aldrich, Saint Louis, MO, USA). The cells were maintained at 37 °C in a humidified atmosphere containing 5% CO_2_ and 95% air, and the culture medium was changed every 2–3 days. After subconfluence, cells were removed from the culture flasks by treatment with 1 mM EDTA (Gibco, Grand Island, NY, USA) and 0.25% trypsin (Gibco) and plated at a density of 5 × 10^4^ cells/well (377 cells/mm^2^) on Control-Ti and Nano-Ti discs in 24-well plates (Corning Inc.) in α-MEM culture medium supplemented with 5 µg/mL ascorbic acid and 7 mM beta-glycerophosphate (Sigma-Aldrich). On days 2 and 4 of culture, recombinant mouse (rm) BMP-7 (5666-BP, R&D Systems, Minneapolis, MN, USA) was added to the culture medium at concentrations of 40 and 200 ng/mL for both groups, based on a previous study [9]. On day 5 of culture, cells were subjected to epifluorescence analysis for the estimation of the cell number and cell proliferation potential, and real-time PCR and Western blotting (WB) were used for mRNA and protein quantification, respectively. The osteoblastic UMR-106 cells (CRL-1661, ATCC) were used for the matrix mineralization assay on day 7 of culture. Similar to the MC3T3-E1 cell culture, they were grown in D-MEM culture medium (Invitrogen) supplemented with 10% fetal bovine serum (Invitrogen) and 1% penicillin–streptomycin (Sigma-Aldrich, Saint Louis, MO, USA). After reaching subconfluence, UMR-106 cells were trypsinized and plated at a density of 1 × 10^4^ cells/well (75 cells/mm^2^) on Control-Ti and Nano-Ti discs in 24-well plates (Corning Inc.) in D-MEM culture medium supplemented with 5 µg/mL ascorbic acid and 7 mM beta-glycerophosphate (Sigma-Aldrich). On days 2 and 4 of culture, the culture medium was supplemented with 40 and 200 ng/mL BMP-7.

### 2.3. Epifluorescence Analyses

#### 2.3.1. Cell Morphology and Cell Counting

On day 5 of culture, MC3T3-E1 cells were fixed in 4% paraformaldehyde in 0.1 M phosphate buffer (PB; pH 7.2) at room temperature (RT). Permeabilization was performed with Triton X-100 at 0.5% in PB. For morphological analysis and cell counting by direct fluorescence, phalloidin conjugated with Alexa Fluor 594, 1:200 (A12381, Invitrogen) was used [24]. Cell nuclei were labeled with 300 nM DAPI (Molecular Probes, Eugene, OR, USA). All the Ti discs were mounted on glass slides (Thermo Fisher Scientific, Waltham, MA, USA), and glass coverslips (Fisherbrand, Thermo Fisher Scientific) were mounted on the Ti disc surface containing the cells using Vectashield anti-fade fluorescence mounting medium (Vector Laboratories, Burlingame, CA, USA). Then, the samples were examined using an Axio Imager 2 fluorescence microscope (Carl Zeiss, Jena, TH, Germany) coupled to an AxioCam MRm digital camera (Carl Zeiss), and images were acquired using the AxioVision 4.8.2 program and processed in Adobe Photoshop CS6 (Adobe Systems, San Jose, CA, USA). Cell counting was performed in three experimental replicates (Ti discs) using a 20× objective by manually counting cell nuclei.

### 2.3.2. Cell Proliferation by Ki-67 Labeling

For the detection of the nuclear protein Ki-67 on day 5 of culture, the fixation and permeabilization steps of MC3T3-E1 cells were the same as described in Section 2.3.1. Fixed and permeabilized cells were first blocked with 5% skimmed milk in PB, and a polyclonal anti-Ki-67 antibody, 1:70 (RP025, Diagnostic Biosystems, Pleasanton, CA, USA), was then used, followed by secondary antibody conjugated with Alexa Fluor 594, 1:200 (A11012, Molecular Probes, Eugene, OR, USA). Between incubations, the samples were rinsed in PB. After DAPI staining and sample mounting, proliferative cells were counted in three experimental replicates (Ti discs) in an Axio Imager 2 fluorescence microscope (Carl Zeiss) using a 20× objective by manually counting Ki-67 positive cells.

### 2.3.3. In Situ ALP Activity by Fast Red Staining

To estimate the in situ ALP activity on day 5 of culture, MC3T3-E1 cultures were first fixed, and cells were then permeabilized as described in 2.3.1. Cultures were washed with PBS and exposed to a 120 mM Tris buffer solution (Sigma-Aldrich) containing 1.8 mM Fast Red TR (Sigma-Aldrich), 0.9 mM naphthol-ASMX phosphate (Sigma-Aldrich), and dimethyl 1:9 (Merck, Darmstadt, HE, Germany) for 30 min in a humidified atmosphere with 5% CO_2_ and 95% air. After DAPI staining and sample mounting, the stained cultures were examined using an Axio Imager 2 fluorescence microscope (Carl Zeiss) with a 20× objective. The acquired images from three experimental replicates were processed in Adobe Photoshop CS6 using the histogram tool to count the red pixels.

### 2.4. mRNA Quantification by Real-Time PCR Analysis

On day 5 of culture, total RNA was extracted using TRIzol LS reagent (Invitrogen). The extracted total RNA was purified using the SV Total RNA Isolation System kit (Promega, Madison, WI, USA). Then, it was quantified at different wavelengths (230, 260, 280, and 320 nm) in the GeneQuant 1300 device (GE Healthcare, Cardiff, WLS, UK) and assessed for its integrity using gel electrophoresis for the intact 28S and 18S ribosomal RNA (Figures S1 and S2). cDNA was synthesized from 1 µg of total RNA by reverse transcription using the High Capacity cDNA Reverse Transcription kit (Applied Biosystems, Foster City, CA, USA). For real-time PCR, TaqMan probes (Applied Biosystems) (Table 1) were used in a CFX96 device (Bio-Rad, Hercules, CA, USA), with a final volume of 10 µL per reaction and 11.25 ng of cDNA. The results were analyzed based on the value of the cycle threshold (Ct), and the normalization and relative quantifications of gene expression were performed by the 2^−ΔΔCT^ method [25]. The data obtained were represented as the fold difference in the expression of the gene normalized to the constitutive gene (*Gapdh*), assigning a value of 1 to each marker in MC3T3-E1 cultures on the Control-Ti surface.

### 2.5. Protein Detection by Western Blotting

On day 5 of culture, MC3T3-E1 cells were processed in an ultrasonic bath (Misonix, Farmingdale, NY, USA), and cell lysis was achieved using RIPA buffer and by means of mechanical shearing. The total protein was then quantified using the micro Lowry assay and the DC™ kit protein assay (Bio-Rad). Briefly, 25 µg of protein from each sample was mixed in 32 µL of lysis buffer with 8 µL of X6 dye, heated, and centrifuged. The proteins were separated by SDS-polyacrylamide gel electrophoresis (10%) and transferred to PVDF membranes (Thermo Fisher Scientific). The membranes underwent three incubations for 1 h at RT: (1) 5% skimmed milk or BSA solution in TBS-T; (2) primary polyclonal anti-phospho-SMAD1/5/9 antibody, 1:1000 (#13820, Cell Signaling Technology Inc., Danvers, MA, USA); primary anti-OPN monoclonal antibody MPIIIB10-1, 1:1000 (AB 2286610, Hybridoma Bank, Iowa City, IA, USA) in BSA solution or 2.5% milk in TBS-T (according to the manufacturer’s recommendations); (3) peroxidase-conjugated secondary anti-IgG1 HRP antibody, 1:2000 (sc-2060, Santa Cruz, Biotechnology, Dallas, TX, USA); secondary anti-IgG HRP peroxidase-conjugated antibody, 1:2000 (#7074s, Cell Signaling Technology Inc.) in BSA solution or 2.5% milk in TBS-T (according to the manufacturer’s recommendations). At the end of each step, the membranes were washed in TBS-T. Then, a wash with chemiluminescent solution (Perkin Elmer, Waltham, MA, USA) was performed, and the membranes were analyzed in the G-Box apparatus (Syngene, Frederik, MD, USA). Then, the membranes were incubated in Re-Blot Plus Mild Solution (Millipore, Burlington, MA, USA) at RT. The primary polyclonal anti-GAPDH antibody was incubated with the membrane at 1:1500 (sc-25778, Santa Cruz Biotechnology, Santa Cruz, CA, USA); the secondary anti-IgG antibody was conjugated to peroxidase at 1:2000 (#7074s, Cell Signaling Technology Inc.) and then incubated with the membrane (according to the manufacturer’s recommendations). The proteins were quantified by the number of pixels of the bands using the programs GeneSys 1.6.9 (Syngene) and GeneTools 4.3.8 (Syngene). The data obtained are represented as fold differences in the expression of the protein normalized to the constitutive protein (GAPDH), assigning a value of 1 to each marker in MC3T3-E1 cultures grown on the Control-Ti surface.

### 2.6. Mineralized Matrix Formation/Calcium Content

On day 7 of culture, UMR-106 cells were rinsed in Hanks’ solution and fixed in 70% ethanol at 4 °C for 60 min and washed in PBS and double-distilled water (dH_2_O). They were stained with 2% Alizarin Red S (ARS, Sigma-Aldrich), pH 4.2, at RT for 15 min, washed with PBS and dH_2_O, and left to dry at RT. ARS extraction was carried out under stirring for 30 min after adding 280 μL of 10% acetic acid to each well. The cell layer was removed, and the samples were then heated to 85 °C, cooled on ice, and centrifuged. From each group, 100 µL of supernatant was added to 40 µL of 10% NH_4_OH. The samples were read on a spectrophotometer (µQuanti, BioTek Instruments Inc., Winooski, VT, USA) using a wavelength of 405 nm. The standard curve was made with the successive dissolution of ARS in ammonium acetate (NH₄CH₃CO₂).

### 2.7. Statistical Analysis

The quantitative data were subjected to two-way ANOVA, followed by Tukey’s post hoc test, at a 5% level of significance using SigmaPlot 11.0 (Statcon, Witzenhausen, Germany). Cell counting, cell proliferation, and in situ ALP activity analyses were carried out in triplicate, whereas the calcium content was determined in quintuplicate.

## 3. Results

### 3.1. Epifluorescence Analyses: Cell Morphology and Cell Counting, Cell Proliferation by Ki-67 Labeling, and In Situ ALP Activity by Fast Red Staining

**Figure 1:**
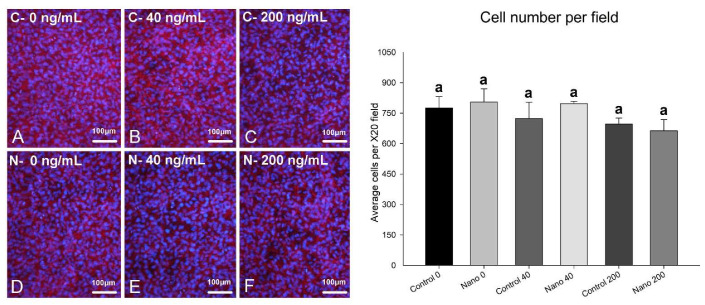
Epifluorescence imaging (left panel) and cell number per field (right panel) of MC3T3-E1 cells on day 5 of culture on Control-Ti (**A**–**C**) and Nano-Ti (**D**–**F**) exposed or not on days 2 and 4 of culture to 40 and 200 ng/mL BMP-7 in the culture medium. (**A**–**F**) Red fluorescence (Alexa Fluor 594 phalloidin) reveals an actin cytoskeleton, and cell nuclei stained with DAPI appear with blue fluorescence. All bars share the same letter and are not significantly different from each other (*p* > 0.05).

**Figure 2:**
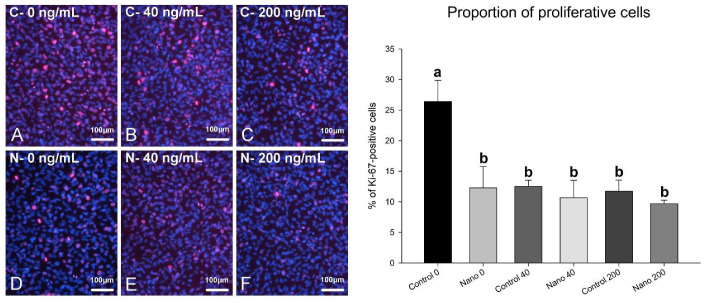
Epifluorescence imaging of Ki-67 labeling (left panel) and proportion of proliferative MC3T3-E1 cells (right panel) on day 5 of culture on Control-Ti (**A**–**C**) and Nano-Ti (**D**–**F**) exposed or not on days 2 and 4 of culture to 40 and 200 ng/mL BMP-7 in the culture medium. (**A**–**F**) Proliferative cell nuclei have red fluorescence (Ki-67+), and all cell nuclei are labeled with DAPI (blue fluorescence). Bars with different letters are significantly different from each other (*p* < 0.05).

**Figure 3:**
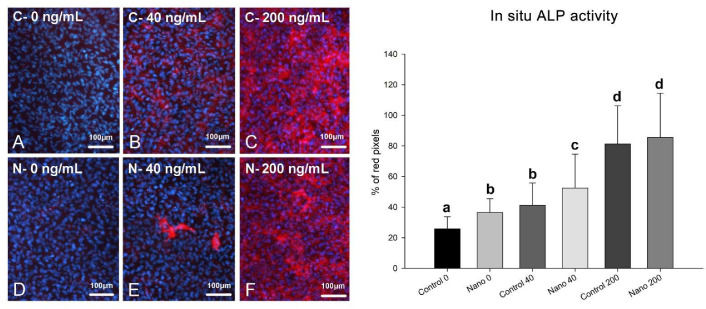
Epifluorescence imaging of Fast Red staining (left panel) and in situ ALP activity (right panel) of MC3T3-E1 cells on day 5 of culture on Control-Ti (**A**–**C**) and Nano-Ti (**D**–**F**) exposed or not on days 2 and 4 of culture to 40 and 200 ng/mL BMP-7 in the culture medium. (**B**,**C**,**E**,**F**) Whole-cell red fluorescence (Fast-Red-stained cells) indicates in situ ALP activity, and cell nuclei blue fluorescence indicates DAPI DNA stain. Bars with different letters are significantly different from each other (*p* < 0.05).

On day 5 of culture, MC3T3-E1 cells were adherent, well spread, and confluent in all the groups, showing multilayer formation and mitotic figures (Figure 1A–F). There was a tendency towards reduced cell numbers when cultures were exposed to 200 ng/mL BMP-7 (Figure 1E,F; bar graph). Ki-67-positive cells were observed in all the groups (Figure 2A–F), not only restricted to mitotic figures, and were fewer in cultures on Control-Ti exposed to BMP-7 and on Nano-Ti exposed or not to BMP-7 in comparison with Control-Ti (Figure 2, compare B–F with A; bar graph). The in situ ALP activity (Figure 3) was detected in all the MC3T3-E1 cultures exposed to BMP-7, revealing polygonal cell shapes, and with greater intensity in cultures grown on Control-Ti and Nano-Ti and exposed to 200 ng/mL BMP-7 (Figure 3, compare C with B and F with E; bar graph). Overall, the MC3T3-E1 cell cultures showed reduced cell proliferation potential and increased in situ ALP activity when exposed to BMP-7 and grown on the Nano-Ti surface.

### 3.2. mRNA Quantification by Real-Time PCR Analysis

**Figure 4:**
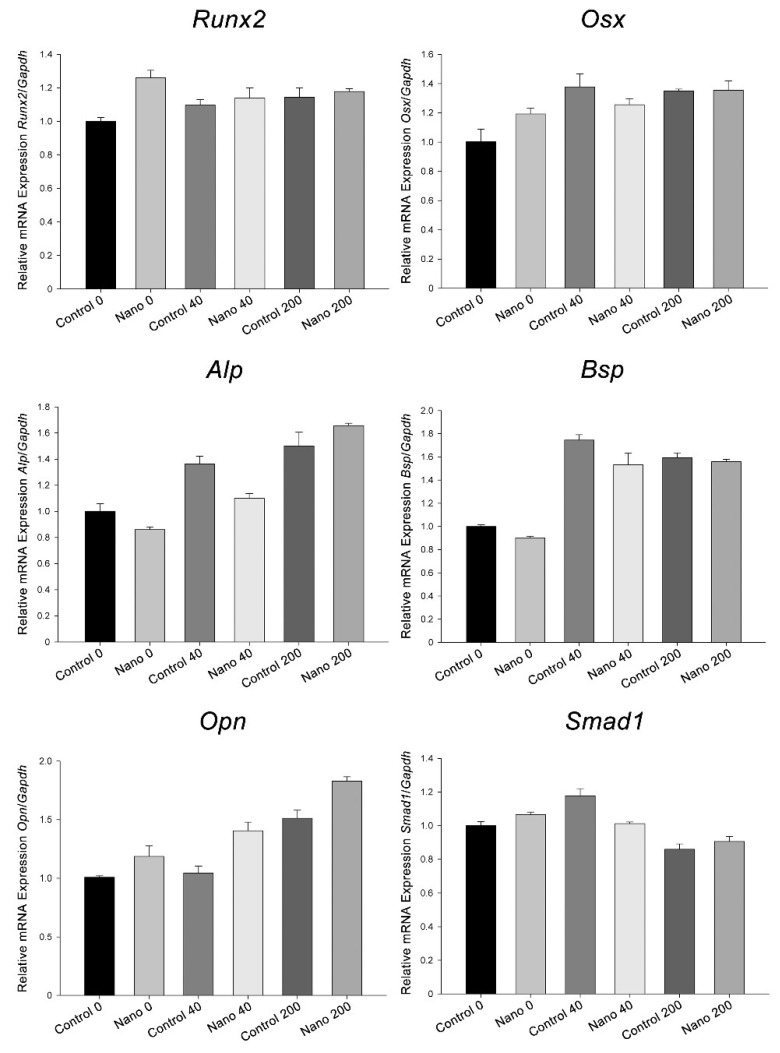
*Runx2*, *Osx*, *Alp*, *Bsp*, *Opn*, and *Smad1* mRNA expression levels normalized to *Gapdh* in MC3T3-E1 cell cultures grown on Control-Ti and Nano-Ti on day 5 of culture, exposed or not on days 2 and 4 of culture to 40 and 200 ng/mL BMP-7 in the culture medium. The bars represent one biological replicate (resulting from the pooling of 16 independent wells/experimental replicates for each group) run in three technical replicates (mean and SD). The mean value of the control group (Control 0) was assigned the value of 1.

The exposure of cultures to BMP-7 and the different Ti surfaces on which they grew altered the mRNA expression levels of the genes evaluated (Figure 4). The results are presented as trends, as no statistical testing was applied due to the pooling of 16 independent experimental replicates for each group. The *Runx2* and *Osx* expression levels were similar on Control-Ti and Nano-Ti when cultures were exposed to BMP-7, and these were higher than those on Control-Ti without any addition of BMP-7 to the culture. The *Alp, Bsp*, and *Opn* expression levels were higher in cultures exposed to BMP-7, except for *Alp* on Nano-Ti, with the 40 ng/mL concentration compared to its control; both *Alp* and *Opn* expression increased gradually with increasing BMP-7 concentration but only *Opn* was higher on Nano-Ti, irrespective of the presence of BMP-7 and its concentration. The *Alp*, *Bsp*, and *Smad1* expression levels were higher in cultures grown on Control-Ti exposed to 40 ng/mL of BMP-7 than on Nano-Ti exposed to the same BMP-7 concentration. *Smad1* levels were decreased in cultures exposed to the 200 ng/mL concentration. In the absence of BMP-7, cultures grown on Nano-Ti showed an increase in *Runx2*, *Osx*, and *Opn* mRNA.

### 3.3. Protein Detection by Western Blotting

**Figure 5:**
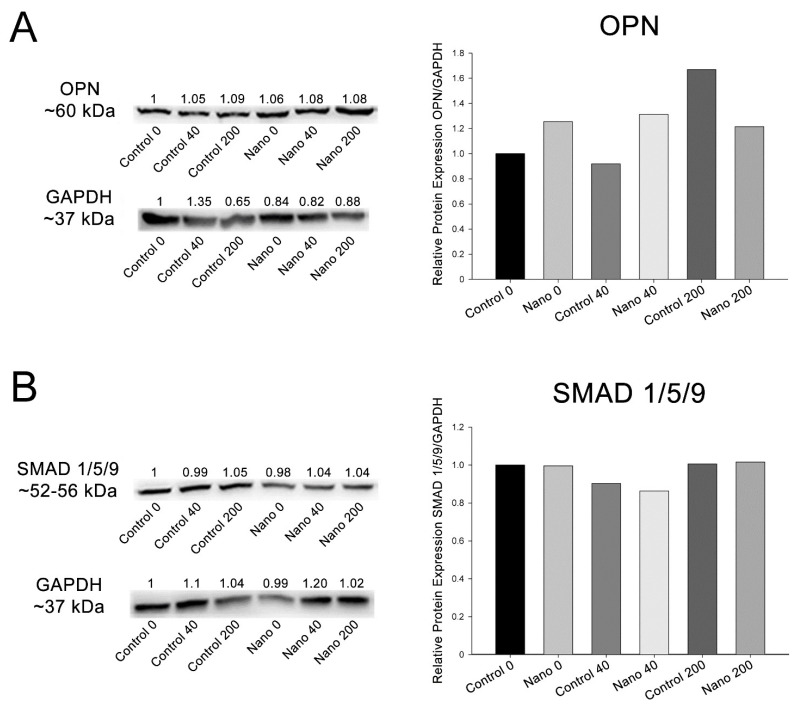
Detection and quantification of OPN (**A**) and the phosphorylated SMAD 1/5/9 complex (**B**) by Western blotting (number of pixels of the protein bands normalized to GAPDH) in MC3T3-E1 cell cultures grown on Control-Ti and Nano-Ti on day 5 of culture, exposed or not at days 2 and 4 of culture to 40 and 200 ng/mL BMP-7 in the culture medium. The bars represent one biological replicate (resulting from the pooling of 20 independent wells/experimental replicates for each group) and one technical replicate. The ratio of the control group (Control 0) was assigned the value of 1.

The results represent one technical replicate from the pooling of 20 independent wells/experimental replicates for each group. OPN was detectable in MC3T3-E1 cultures in all the groups, showing higher amounts on Nano-Ti compared to its controls except for at the 200 ng/mL concentration (Figure 5). The phosphorylated SMAD 1/5/9 complex was also detectable in all the groups but in lower amounts when cells were exposed to the 40 ng/mL concentration on both Ti surfaces (Figure 5).

### 3.4. Mineralized Matrix Formation/Calcium Content

**Figure 6:**
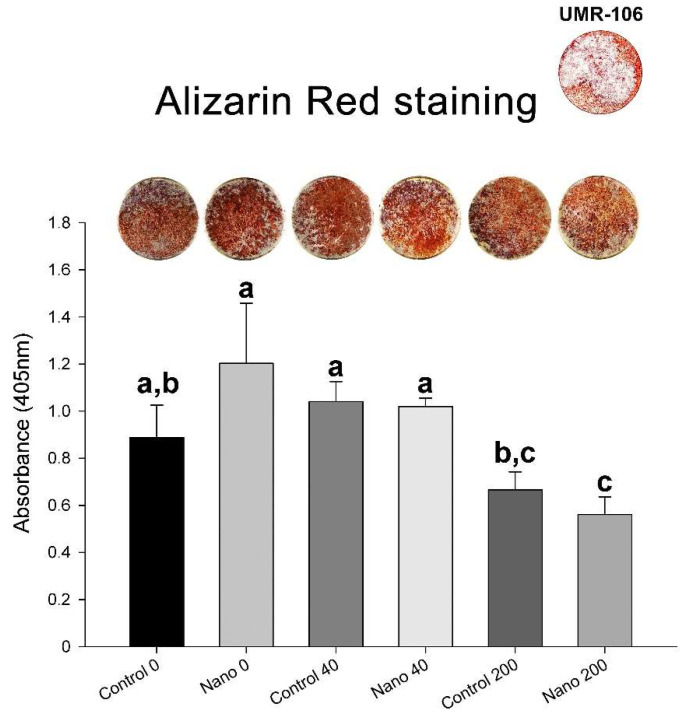
Macroscopic imaging and biochemical analysis (calcium content) of Alizarin-Red-stained mineralized matrix formation of UMR-106 cell cultures grown on Control-Ti and Nano-Ti on day 7 of culture, exposed or not on days 2 and 4 of culture to 40 and 200 ng/mL BMP-7 in the culture medium. Bars that share one letter are not significantly different from each other (*p* > 0.05).

Qualitatively, the UMR-106 cultures exhibited an osteogenic phenotype in all the groups (Figure 6). Quantitatively, the results of the Alizarin Red staining showed no significant differences between the groups with the absence of BMP-7 or its 40 ng/mL supplementation. However, 200 ng/mL BMP-7 supported lower mineralization for both Control-Ti and Nano-Ti (Figure 6).

## 4. Discussion

The present study partially refutes the null hypothesis that BMP-7′s effects are not modulated by the Ti surface nanotopographic features with which osteoblastic cells interact. Important in vitro osteogenic effects were observed on Control-Ti in terms of cell proliferation potential, gene expression (*Runx2*, *Osx*, *Alp*, *Bsp*, *Opn*, and *Smad1*), and in situ ALP activity. Regarding Nano-Ti, the stimulus for osteogenic differentiation was limited to an increase in *Alp*, *Bsp*, and *Opn* expression and in situ ALP activity, which occurred with the exposure of cell cultures to 40 ng/mL BMP-7. Importantly, the analysis of matrix mineralization revealed that exposure to 40 ng/mL BMP-7 did not alter the osteogenic potential of cultures grown on both Ti surfaces, whereas 200 ng/mL reduced it by about 40%.

Over the last few decades, one of the main objectives of bone implantology has been stimulating osteogenic differentiation at the bone–metal interfaces of osseointegrated implants by means of varied strategies, particularly for their use in sites of lower bone density [26,27,28]. In the present study, we opted to evaluate the impact of the extracellular availability of exogenous BMP-7 at two different concentrations in the culture medium during the initial proliferation phase of pre-osteoblastic MC3T3-E1 cells [29] on key osteogenic parameters when cells grew on Ti with distinct surface topographies at the nanoscale. Previous studies by our group revealed that osteogenic cells showed greater responsiveness to exogenous BMP-2 and greater production of endogenous BMP-2 when grown on Nano-Ti [12,20]. The concentrations of 40 and 200 ng/mL were defined based on other cell culture studies that used recombinant BMP-7 at concentrations ranging from 10 to 400 ng/mL, which evokes stimulatory and/or inhibitory effects on osteogenic differentiation. The stimulatory effects were predominant up to 200 ng/mL, after which the results are controversial [8,9,10,30,31], with a report of inhibitory effects between 200 and 400 ng/mL [9].

The in vitro conditions used here showed that the effects of BMP-7 on MC3T3-E1 cells were more remarkable on Control-Ti than on Nano-Ti, similar to those observed elsewhere for polystyrene [9]. Importantly, the Nano-Ti per se (with no BMP-7 added to the culture medium) promoted proliferative activity; *Runx2*, *Osx*, and *Opn* mRNA expression; OPN protein expression; and mineralized matrix formation compared with Control-Ti, a pro-osteogenic in vitro effect that has been described previously [19,24,32]. Thus, the extra stimulus with the addition of BMP-7 to the culture medium was more effective on cells that were growing on Control-Ti than on Nano-Ti, which agrees with the concept that Ti surfaces that are flat/smooth at the micron and nano scales can also benefit from functionalization with bioactive molecules [33]. However, when developing strategies for surface functionalization, the important advantages of using micro- and/or nanostructured Ti surfaces should be considered. In fact, the extension of the surface area has a direct effect on the number of grafted molecules, and the presence of hydroxyl and other functional groups, which often occurs on etched titanium surfaces, supports the functionalization of organic molecules by electrostatic or covalent linking [34]. Although Nano-Ti may not require the same level of surface functionalization as Control-Ti and, in some cases, even none at all, this is an important tool that must be applied when specific cellular functions are needed (reviewed in [35,36]).

Among the five classical osteoblast markers used here, only *Opn* showed a proportional increase in mRNA expression with an increase in the BMP-7 concentration for both Control-Ti and Nano-Ti, although this did not entirely correspond with the WB results at the same time point of culture. In primary cultures of fetal rat calvarial cells [37], BMP-7 stimulates a rapid induction of osteopontin (OPN) mRNA in confluent cultures enriched in pre-osteoblastic cells, whereas a weak induction of OPN is observed when BMP-7 is added to nodule-forming cultures (enriched in osteoblastic cells). In a recent previous report [38], the transfection of rat dental follicle cells with a recombinant adenoviral vector encoding BMP-7 (Ad-BMP7) resulted in its overexpression, which eventually induced osteogenic differentiation by increasing ALP activity and OPN protein levels. Similar results at the molecular level were observed for human dermal-derived CD105+ fibroblast cells that were induced to osteogenic differentiation through adenovirus-mediated recombinant BMP-7 expression [39]. Despite the unequivocal importance of OPN expression for the acquisition of the osteogenic phenotype and the control of matrix mineralization [40], OPN’s overexpression in MC3T3-E1 cells could exert an inhibitory effect on osteoblastic differentiation, as demonstrated previously [41]. Moreover, as the diverse biological functions of OPN are modulated by plasma proteases such as thrombin and plasmin [42,43], any further in vitro evaluation of the initial interactions of osteoblastic cells with BMP-7-functionalized Ti surfaces should ideally include a preformed physiological blood clot on the material surface (ex vivo model).

In order to assess the impact of BMP-7 on the MC3T3-E1 cell signaling pathway, quantification of the mRNA for *Smad1* and the detection of the phosphorylated SMAD 1/5/9 complex were performed. The cytoplasmic expression of SMADs is increased with the activation of BMP receptors, and their translocation to the nucleus results in the transcription of critical target genes, such as *Runx2*, for the induction of osteoblast differentiation [44]. In the present study, there was not a direct correspondence between the presence of BMP-7 in the culture medium and *Smad1* expression levels in MC3T3-E1 cells except for cultures grown on Control-Ti exposed to 40 ng/mL of BMP-7, which, however, did not correspond to the amount of phosphorylated SMAD 1/5/9 at the same time point. Although it is well accepted that the relationship between transcription and translation is complex and that there may be no correlation between the expression of an mRNA and the protein it encodes (discussed in [45]), the effects of BMP-7 on *Smad1* expression by osteoblastic cells should be further investigated more comprehensively.

To evaluate the acquisition of the osteogenic phenotype on Control-Ti and Nano-Ti with exposure to either 40 or 200 ng/mL of BMP-7, we opted to use the UMR-106 cell line [46], the cells of which are in a more advanced stage of osteoblastic differentiation than MC3T3-E1 cells [46,47] and capable of producing a collagen matrix that undergoes mineralization in a shorter culture period [48]. This strategy allowed us to maintain the same protocol to expose UMR-106 cells to BMP-7 as the one applied to MC3T3-E1 cells. Importantly, the mineralized matrix secreted by UMR-106 cells has been comprehensively studied, and its mineral phase is similar to that of bone in terms of its crystal size and Ca/P ratio [48]. The results revealed no direct benefit for the amount of bone-like matrix formation except for a slight tendency, in the order of 10%, to greater mineralization on Control-Ti when cells were exposed to BMP-7 at 40 ng/mL. The reduction in the osteogenic potential of the cultures on both Ti surfaces was remarkable with the 200 ng/mL concentration. These results are not surprising when considering the in vitro system used, in which there is no renewal of the cell population over the culture period. BMP-7 reduces the proliferative capacity potential of MC3T3-E1 cells, as observed in the present study and elsewhere [9], which results in a tendency towards smaller cell populations with an increase in BMP-7 concentration in the culture medium. It is reasonable to assume that this effect would also occur in UMR-106 cells, thus limiting the extent of mineralized matrix formation. Despite that, these effects must be attenuated in in vivo biological models of bone repair, in which cell renewal occurs; the effects of BMP-7 availability might result in benefits for the repair of bone in contact with biomaterials [49,50,51].

## 5. Conclusions

In conclusion, the effects of the extracellular availability of BMP-7 on osteogenic differentiation in vitro might vary substantially as a function of the surface topography at the nanoscale on which cell cultures grow, the concentration of growth factor in the culture medium, and the stage of osteoblastic cell differentiation. The presence of rmBMP-7 in the culture medium alters the expression profile of osteoblast markers in pre-osteoblastic cells, indicative of the acquisition of a more advanced stage of osteoblastic differentiation at the concentrations used. This occurs with less impact on the nanostructured Ti and without resulting in higher mineralized matrix production by differentiated osteoblasts on both studied surfaces. Strategies for the development of metallic implants functionalized with BMP-7 (and probably with other bioactive molecules) should take into consideration the release profile during the repair process based on the concentration used for functionalization and the physical–chemical features of the implant surface, with the aim of ensuring specific cell behavior and/or generating a proper tissue phenotype.

## Figures and Tables

**Table 1:** TaqMan probes used in real-time PCR analysis.

Genes	TaqMan Probe
*Runx2*	Mm00501584_m1
*Osx*	Mm04933803_m1
*Alp*	Mm00475834_m1
*Bsp*	Mm00492555_m1
*Opn*	Mm00436767_m1
*Smad1*	Mm00484723_m1
*Gapdh*	Mm99999915_g1

## Supplementary Materials

The following supporting information can be downloaded at: https://www.mdpi.com/article/10.3390/biomimetics7030136/s1. Figure S1: RNA integrity—Electrophoresis file run summary; Figure S2: RNA integrity—Electropherogram summary.
