# Supplementary figures and images for: Effects of rmBMP-7 on Osteoblastic Cells Grown on a Nanostructured Titanium Surface
Source: Biomimetics (Basel). 2022 Sep 16;7(3):136. doi: 10.3390/biomimetics7030136 (PMC9496167; doi:10.3390/biomimetics7030136)

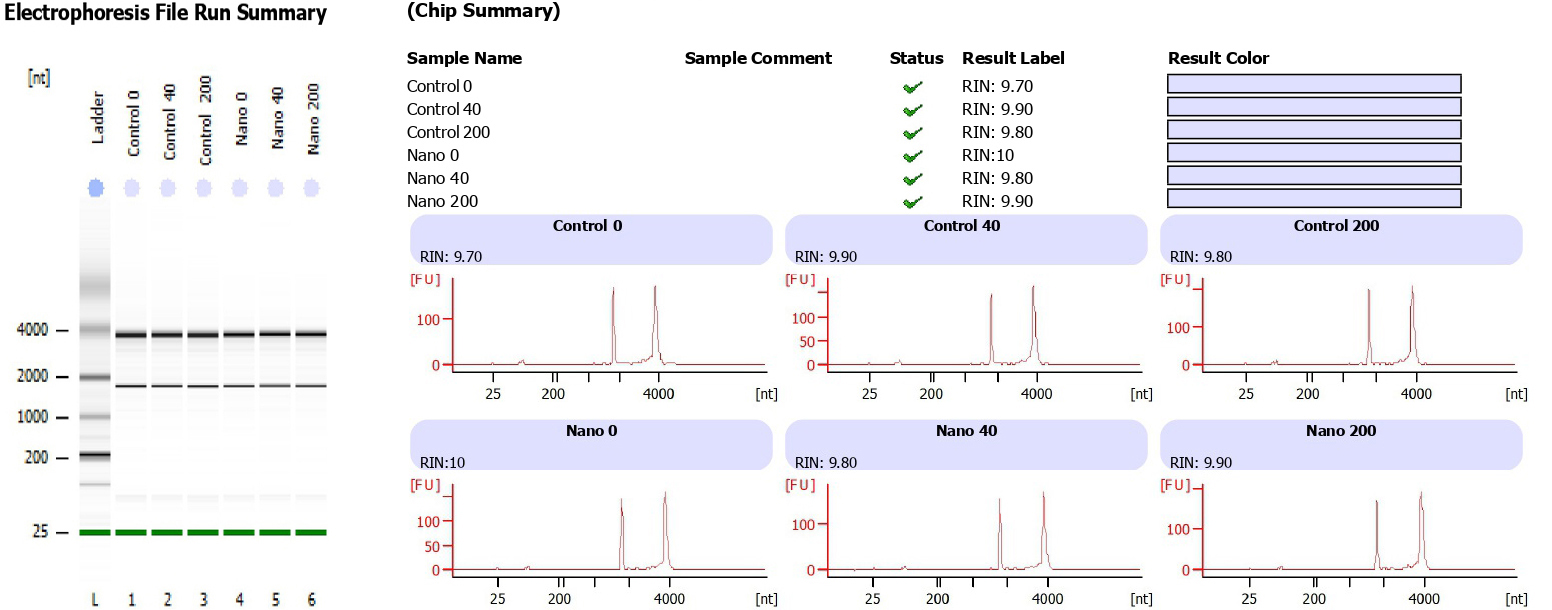

Supplement: Supplementary file 1 [file biomimetics-07-00136-s001.zip › Figure S1 - RNA integrity - Electrophoresis file run summary.jpg]

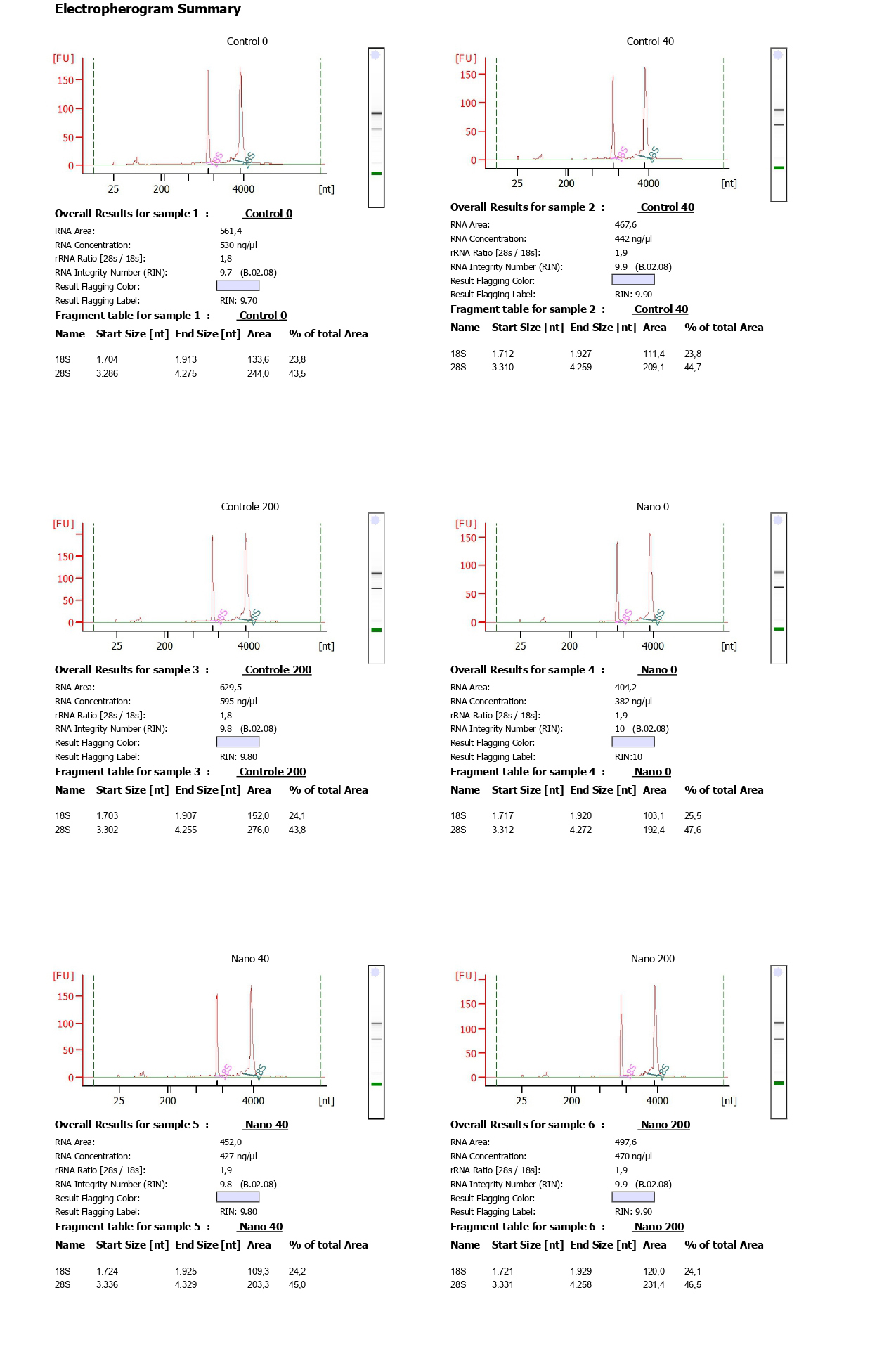

Supplement: Supplementary file 1 [file biomimetics-07-00136-s001.zip › Figure S2 - RNA integrity - Electropherogram summary.jpg]
